# Inbreeding and Genetic Differentiation Among Geographic Populations of *Lactarius hatsudake* in Southwest China

**DOI:** 10.3390/jof11060438

**Published:** 2025-06-08

**Authors:** Kuan Zhao, Mingwei Mao, Xianghua Wang, Jianping Xu

**Affiliations:** 1 Key Laboratory of Natural Microbial Medicine Research of Jiangxi Province, College of Life Science, Jiangxi Science and Technology Normal University, Nanchang 330013, China; key1989@126.com (K.Z.); MMW0403@163.com (M.M.); 2 Key Laboratory for Plant Diversity and Biogeography of East Asia, Kunming Institute of Botany, Chinese Academy of Sciences, Kunming 650201, China; 3 Department of Biology, McMaster University, Hamilton, ON L8S 4K1, Canada

**Keywords:** milk cap mushroom, simple sequence repeat, divergence, gene flow, inbreeding

## Abstract

*Lactarius hatsudake* is an economically important wild edible fungus in sub-tropical and temperate Asia. At present, little is known about its genetic diversity, mode of reproduction, and population structure in natural environments. In this study, we collected 102 specimens from eight geographic locations across three provinces in southwest China. Five simple sequence repeat markers that demonstrated high polymorphism were developed and used to analyze the patterns of genetic variations within and among the eight geographic populations. Analyses of molecular variance revealed that 60% of the observed genetic variation occurred among populations, with the remaining 40% attributable to within populations, while inter-provincial variation was nonsignificant. Combined analyses based on discriminant analysis of principal components, STRUCTURE, and the unweighted pair group method with arithmetic mean identified two distinct genetic subpopulations with each genetic subpopulation showing a wide geographical distribution, a result consistent with ancient divergence and recent gene flow within this species in southwest China. Interestingly, excess homozygosity was observed at most loci in almost all of the eight geographic populations, consistent with inbreeding being common for this species in nature. Together, our results revealed the genetic diversity, mode of reproduction, and geographic structuring of this important wild mushroom in southwest China.

## 1. Introduction

**Figure 1:**
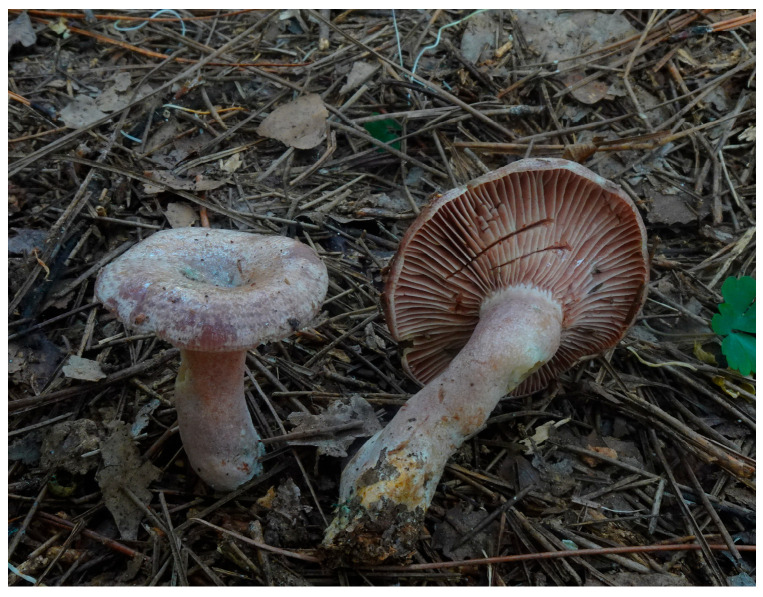
The basidiocarps of *Lactarius hatsudake* in their natural habitat.

*Lactarius hatsudake,* the common milk cap mushroom, belongs to the fungal family Russulaceae in the order Agaricales. It is an endemic edible mushroom in Asia and common in the southern regions of China [1,2]. The basidiocarps of *L. hatsudake* in its natural habitat are shown in Figure 1. As an obligate ectomycorrhizal fungus, *L. hatsudake* is only found in forests of pine trees, such as *Pinus densiflora*, *P*. *massoniana*, and *P*. *yunnanensis* [3,4,5]. The fruiting bodies of *L. hatsudake* are rich in essential nutrients such as amino acids, minerals, and polysaccharides [6,7]. Specifically, a total of 17 amino acids (including 7 essential amino acids) and 7 mineral elements have been reported in the fruiting bodies of *L. hatsudake*, with phenylalanine, leucine, iron (Fe), manganese (Mn), and zinc (Zn) concentrations notably exceeding those found in other commonly consumed mushroom species [6,7]. Indeed, it is one of the wild edible mushrooms favored by millions of people in east Asia. It serves not only as a delicious food but has also shown great potential for regulating the human intestinal microbiota and inhibiting tumors [7].

Despite its economic importance, our knowledge about its basic biology remains limited. At present, the mating system of *L. hatsudake* is not known. However, because it produces mushrooms and basidiospores, the species likely follows the typical basidiomycete life cycle, which involves both sexual and asexual phases [8,9,10]. Sexual reproduction in basidiomycete mushrooms is characterized by the formation of fruiting bodies that can undergo meiosis and produce basidiospores. The basidiospores disperse into the environment and when conditions are suitable, germinate to form haploid mycelia. Basidiomycete fungi can accomplish a sexual reproduction cycle through a diversity of reproductive strategies, including homothallism, pseudo-homothallism, and heterothallism. In homothallic basidiomycete species, each basidiospore has the potential to complete the whole sexual reproductive life cycle by itself without the need for mating. In such cases, there would be limited genetic variation within individual fruiting bodies, and except for gene duplication that would generate paralogs, most genes should be homozygous within most fruiting bodies. At the population level, alleles at the same locus would be expected to deviate significantly from Hardy–Weinberg equilibrium (HWE) [11]. In addition, alleles at different loci would be expected to show significant linkage disequilibrium [11,12].

In heterothallic species, haploid mycelia with different mating types are required for successful mating and dikaryon formation, followed by fruiting body formation—hallmarks of sexual reproduction in most heterothallic basidiomycetes. Sexual compatibility in heterothallic fungi may be controlled by one genomic region (i.e., one mating type locus; bipolar heterothallism) or two genomic regions (i.e., two mating type loci, tetrapolar heterothallism) [8,13]. In general, the heterothallic mating system facilitates outcrossing (mating between genetically distinct individuals) and promotes genetic diversity within individuals and populations. For example, the heterothallic mating system experiences negative frequency-dependent selections, where rare mating types in populations tend to have higher mating success than abundant mating types, contributing to the maintenance of many mating types and the overall genetic variations within the populations of many of these species in nature. However, inbreeding, either through self-fertilization or mating between closely related individuals, can also happen in heterothallic mushrooms. Inbreeding reduces genetic variations within individuals, as shown by the high prevalence of homozygosity [14].

The complex interplay between mating systems, outcrossing, inbreeding, and selection pressures directly shapes the genetic diversity and structure of mushroom populations. Deciphering these genetic patterns requires robust molecular tools capable of characterizing variations across genomes and populations. Diverse molecular markers have been employed to analyze the populations of mushroom-forming fungi, as well as other types of organisms. Each type of marker has its advantages and limitations. For example, Random Amplified Polymorphic DNA (RAPD) markers enable rapid, low-cost screening without prior sequence knowledge, but exhibit low reproducibility and dominant inheritance, restricting precise heterozygosity estimation [15,16]. Ribosomal DNA (rDNA) ITS/IGS regions serve as the consensus fungal DNA barcodes for taxonomic identification but have limited or no intraspecific variability [17]. Amplified Fragment Length Polymorphisms (AFLPs) generate genome-wide profiles for clonal discrimination, yet suffer from technical complexity, and similar to RAPD, have dominant inheritance biases, restricting precise heterozygosity estimation [18]. Simple Sequence Repeats (SSRs) offer high polymorphism and codominant inheritance but require prior genome sequencing for primer design [19,20]. Inter-Simple Sequence Repeats (ISSRs) provide cost-effective, sequence-independent screening, but yield dominant markers unsuitable for heterozygosity analysis [21]. Sequence-Related Amplified Polymorphisms (SRAPs) target functional gene regions with high reproducibility but exhibit lower polymorphism than SSRs [22]. Single Nucleotide Polymorphisms (SNPs) enable ultra-high resolution but incur substantial sequencing costs for non-model species [23]. Emerging techniques, like RAD-seq, complement these markers, yet face limitations including high cost and complex bioinformatics requirements [24]. Marker selection must align with the study’s goals, the target species’ genomic resources, and the budgetary limitations. When a reference genome exists, SSRs remain particularly well-suited for fine-scale population structure analysis.

In this study, we are interested in the mode of reproduction of *L. hatsudake* in nature. In addition, we are interested in the relationships among geographic populations of this species in southwest China. *Lactarius hatsudake* is endemic in southwest China [1,17]. This region is characterized by high mountain ranges, deep valleys, and fragmented microhabitats, including the large Hengduan mountain ranges [25]. Mountainous topography can limit spore dispersal, isolate populations, and promote genetic differentiation among the isolated geographic populations. However, mycophagous animals, such as rodents, insects, and anthropogenic factors, such as mushroom picking and trading, may counteract the potential genetic isolations by geographic barriers [26,27]. Major anthropogenic factors for this and many other ectomycorrhizal mushrooms are logging, habitat fragmentation, and the recent reforestation of the host pine trees, such as *Pinus yunnanensis*, since the 1950s. For instance, the widespread reforestation of *Pinus yunnanensis* in southwest China has likely contributed to the genetic homogeneity of *P. yunnanensis* populations, where low population differentiation (*Fst* = 0.045) was found across broad geographic areas in this region, with no evidence of genetic isolation by geographic distance, a stark contrast to the natural dispersal constraints imposed by topography and short-range seed dispersal [28]. In addition, since *L. hatsudake* has been found in forests dominated by different pine trees in different regions, it is not known whether host tree species influence the population genetic structure of *L. hatsudake*.

A previous study analyzed 41 fruiting bodies of *L. hatsudake* from six locations in four provinces (Hunan, Guangxi, Guizhou and Yunnan) in southern China [17]. In this study, the authors obtained 621 bp DNA sequence at the internal transcribed spacer (ITS) regions of the ribosomal RNA gene cluster from each mushroom and reported that the 41 samples belonged to 18 ITS sequence types. While most of the ITS sequence types showed restricted geographic distributions, one type was broadly distributed and found in five of the six local populations, consistent with long-distance gene flow. Indeed, the analyzed geographic populations showed no statistical evidence for genetic differentiations, with an overall *Fst* value of about 0.04 (pairwise *Fst* values ranging between 0.0092 and 0.093). However, several reasons make those conclusions tentative. First, only a single marker was analyzed; thus, the allelic relationships among markers cannot be assessed. Second, the ITS belongs to a highly repeated region of the genome and is subjected to concerted evolutionary pressure, making it less variable than most other parts of the genome. Consequently, ITS sequences have low discriminate power among individuals within a species [29]. Third, the repetitive nature of the ITS locus makes it difficult to identify potential heterozygosity within individual mushroom samples and its potential mode of reproduction in nature [29]. Lastly, a relatively small number of strains were analyzed.

To overcome these problems and to critically evaluate the mode of reproduction and population structure of this species in nature, we developed five highly polymorphic SSR markers based on the whole-genome sequences of *L. hatsudake* and used those markers to analyze 102 fruiting bodies from eight discrete geographic locations in southwest China. Our analyses revealed evidence for inbreeding within all local populations and significant genetic differentiation among most of the geographic populations analyzed. We discuss the implications of these results on the population history and conservation of genetic resources for this important wild edible mushroom.

## 2. Materials and Methods

### 2.1. Sample Collection and DNA Extraction

The fruiting body samples of *L. hatsudake* were collected from eight geographic sites in southwest China. For each site, information about its location, sample size, range of altitude, and dominant host plant, is listed in Table 1.

**Figure 2:**
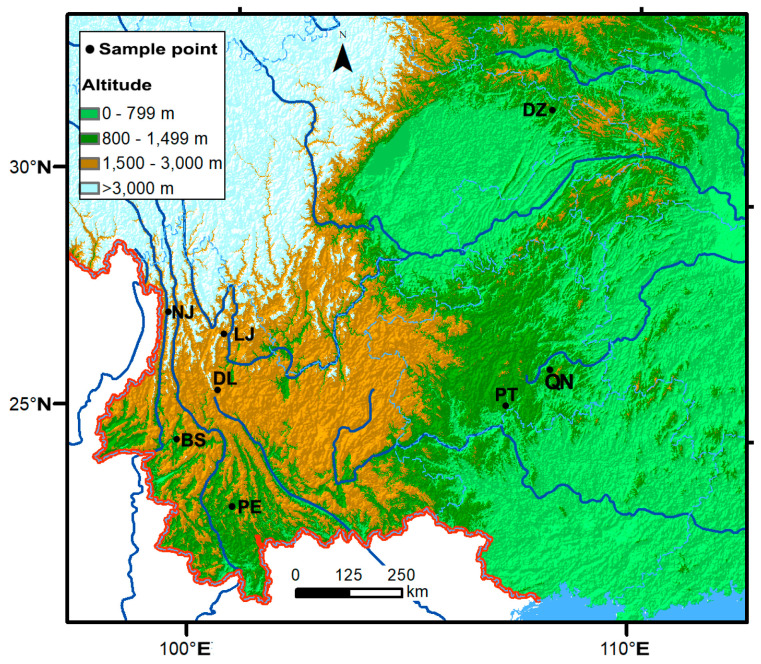
Geographical distributions of the eight collection sites of *Lactarius hatsudake* populations in southwest China. The sampling sites are represented by black dots, and details of their abbreviated codes are shown in Table 1.

The geographic locations of the eight sites are shown in Figure 2 (map generated with ArcGIS v10.0, https://www.esri.com/en-us/arcgis/products/arcgis-for-personal-use/overview, accessed 8 February 2025). Within each site, the fruiting bodies were collected from the natural habitats according to the collection methods introduced in the previous literature and supplemented by local mushroom market in the years of 2022 and 2023. Fruiting bodies were identified for the species based on their morphology and microscopic features [1]. The genomic DNA was extracted from each fruiting body using the modified CTAB method [30].

### 2.2. Discovery and Development of SSR Markers

To identify polymorphic SSR markers, we first downloaded the whole-genome sequence of *L. hatsudake* strain 109 from GenBank (https://www.ncbi.nlm.nih.gov/datasets/genome/GCA_021525005.1/, accessed on 15 October 2024). Individual contigs were uploaded onto the online MIcroSAtellite identification (MISA) tool [31], and over 200 di- and trinucleotide SSR loci were found. PCR primers for these SSR loci were generated with the integrated Primer3 tool [32]. The generated primers were then selected using the following criteria: length range, 18–22 nucleotides; PCR product size, 100–250 bp; melting temperature (Tm), 58–60 °C; and GC content, 50–60%. The screening identified 24 putative SSR loci along with their primer pairs for further testing. Specifically, these primer pairs were synthesized and tested for evidence of polymorphism among 16 mushroom specimens, including two specimens from each of the eight local populations. Based on PCR success rate, reproducibility, and polymorphism, five SSR markers were selected to analyze all the 102 specimens collected for this study. The information of the five primers and their locations in the genome are presented in Table 2.

For SSR genotyping, each PCR reaction mixture consisted of 1 µL of template DNA, 0.5 µL each of forward and reverse primer (5 pmol), 5 µL of 2 × Taq Master Mix (Sangon Biotech Shanghai Co. Ltd., Shanghai, China), and 5 µL of distilled water. PCR reactions were performed as follows: 95 °C for 3 min, 35 cycles of 95 °C for 30 s, 54 °C for 30 s, 72 °C for 30 s, and finally, 72 °C for 10 min. The SSR fragments at the five loci were separated using capillary electrophoresis at Sangon Biotech Shanghai Co. Ltd., China and then analyzed using the SSR genotyping software GeneMarker v.2.9.0 [33] to determine the fragment length for each marker locus in each specimen. The polymorphic information content (PIC) values of the five loci were calculated separately by Cervus [34] to evaluate their polymorphism level.

### 2.3. Allelic and Genotypic Diversities

Allelic diversity, including the number of unique alleles (Na), the effective number of alleles (Ne), and unbiased allelic diversity (uH), was assessed for each of the five SSR loci across the eight populations using the GenAlEx 6.5 program integrated with Excel [35,36]. The unbiased allelic diversity reflects the likelihood that two randomly selected alleles at each locus from the population will be different, while the unbiased genotypic diversity indicates the probability that two randomly drawn individuals will possess distinct multilocus genotypes (MLGs). The diversity indices range from 0 to 1, where a value of 0 signifies no variation, indicating that all samples share the same allele at a specific locus or the same MLG in the population, whereas a value of 1 indicates high genetic variation, meaning that any two randomly selected alleles or individuals will differ. To evaluate whether the populations significantly differed in their mean uH and mean Ne, two-tailed Student’s *t*-tests were performed.

### 2.4. Mode of Reproduction

To assess the mode of reproduction of *L. hatsudake* in southwest China, we conducted three tests. In the first, we looked for evidence of heterozygotes. The presence of heterozygotes across individual mushrooms would be inconsistent with homothallism but instead suggests pseudo-homothallism or heterothallism. In the second, we conducted a HWE test to assess the relationships among alleles at each locus. For this test, the observed frequencies of genotypes at each locus were compared with those expected based on random mating. Significant departures of the observed genotype frequencies from the expected levels indicate non-random mating in natural populations. Furthermore, an excess of observed homozygotes over the expected levels is consistent with inbreeding [11]. In the third, we investigated the relationships between genotypes at different loci for evidence of genotypic disequilibrium. Significant associations among genotypes at different loci would suggest genetic linkage and non-random associations among loci [11]. Both the HWE and genotypic disequilibrium tests were conducted using GenAlEx 6.5 [35,36].

### 2.5. Relationships Among Geographic Populations in Southwest China

The *Rst* statistic developed specifically for SSR markers was calculated by GenAlEx 6.5 program to evaluate genetic differentiation among geographic populations. The analysis of molecular variance (AMOVA) was also performed using GenAlEx to assess the contributions of geographic separation to the total observed genetic variations among populations [37]. Two hierarchical levels of analyses were conducted for the 102 samples. One level was among eight local populations (patches), and the second level was between the three regional populations (three provinces, Table 1). To visualize the distributions and genetic relationships among the MLGs within and between the local populations of *L. hatsudake*, a minimum spanning network (MSN) tree was constructed using the R package *poppr* [38] (https://github.com/grunwaldlab/poppr/, accessed 15 January 2025). The network was based on Bruvo’s genetic distances among strains, and the minimum edge genetic distance was set to 0.05.

### 2.6. Identification of Genetic Clusters Within Our Population Sample

The unweighted pair group method with arithmetic mean (UPGMA) is an agglomerative hierarchical clustering method which combines the nearest two clusters or elements into a higher-level cluster step-by-step, and the distance between the new cluster and any other cluster is calculated as the arithmetic mean distance between elements in different clusters [39]. A UPGMA cluster analysis was performed based on Nei’s unbiased genetic distance matrix with MEGA X [40] and then visualized using the Interactive Tree of Life (iTOL) (https://itol.embl.de/, accessed on 20 January 2025) [41].

To investigate whether there are distinct genetic clusters within our population, we used STRUCTURE 2.3.4 software with a Bayesian approach [42,43,44,45]. We tested models for K-values ranging from 1 to 8 and conducted 10 independent runs for each K value. Each run included an initial burn-in period of 200,000 iterations followed by 1,200,000 MCMC iterations. ΔK values were calculated using the method described by Evanno et al. 2005 [42].

In addition, a multivariate analysis called discriminant analysis of principal components (DAPC), implemented using the *adegenet* package in R [46] (http://adegenet.r-forge.r-project.org/, accessed through 20 January 2025), was used to cluster MLGs in relation to their geographic origins. In this analysis, DAPC first transformed the data using principal component analysis (PCA) to reduce the number of variables, allowing the retention of the variables that make the greatest contribution to the variation within the dataset. This was followed by discriminate analysis (DA) to cluster the MLGs by optimizing the between-group variation and reducing the within-group variation.

## 3. Results

### 3.1. Allelic and Genetic Diversity

The SSR genotypes were obtained for all 102 samples and their detailed allelic and genotype information for each sample is presented in Supplementary Table S1. Our analyses showed that all five markers were polymorphic for all eight local populations. The PIC values of the five loci detected by Cervus were 0.893, 0.923, 0.870, 0.837, and 0.790, respectively, with an average value of 0.863.

Among the eight local populations, the DZ population in Sichuan Province had the highest number of unique and effective alleles, whereas the QN population in Guizhou Province had the highest amount of unbiased allelic diversity (Table 3). However, the differences among the eight local populations in the allelic diversity indicators were statistically not significant (two-tailed Student’s *t*-tests, *p* > 0.05 in all tests). Together, the results indicated similar level of allelic diversities among the eight discrete local populations of *L. hatsudake* in southwest China.

### 3.2. Genotypic Diversity and Allelic Associations

As shown in Supplementary Table S1, there was a high multilocus genotypic diversity in all eight local populations. Among the 102 analyzed fruiting bodies, 100 unique MLGs were identified, with only two MLGs being represented by two fruiting bodies each while the remaining 98 MLGs were each represented by one specimen. In addition, all five loci showed heterozygosity, and all strains had at least one of the five loci being heterozygous. The abundant heterozygosity, coupled with the high multilocus genotypic diversity in both the total population and within each local population, was inconsistent with the homothallic life cycle but consistent with heterothallism for this species.

To investigate whether the observed genotype frequencies deviated from expected genotype frequencies based on random mating, we conducted a HWE test for each locus for each population. Our analyses rejected the null hypothesis of HWE for all the five loci in the four local populations (BS, DL, DZ, and PT) with more than 10 specimens (Table 4). However, due to the small sample size in the remaining four local populations, the results of the HWE test were tentative, with mixed statistics among the loci in three of the four local populations. Interestingly, aside from two loci (Lh5 and Lh14) in the DZ local population, all other loci-local population combinations showed evidence of heterozygous deficiency. Together, the results are consistent with frequent inbreeding for this species in nature.

A genotypic disequilibrium test between all pairs of loci across all local populations failed to reject the null hypothesis of random genotype associations (*p* > 0.1 in all cases, detailed results not shown). This result is consistent with the frequent recombination among loci within the genome of this species in nature.

### 3.3. Genetic Relationships Between Strains

**Figure 3:**
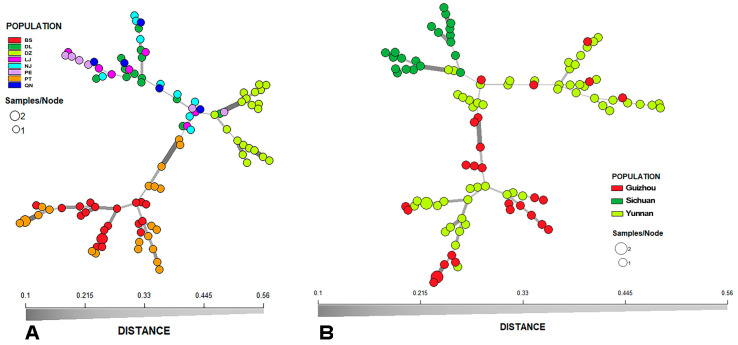
Minimum-spanning network showing the genetic relationship between all 100 MLGs from eight local populations (**A**) and three regional populations (**B**). Each circle represents one MLG. The genetic distance between MLGs was calculated using Bruvo’s genetic distance.

Our analyses revealed that only 2 of the 100 MLGs appeared twice. Both shared MLGs were shared by fruiting bodies from the same local population: one shared MLG was between two strains from the BS local populations in Yunnan Province, and the other shared MLG was between strains from the PT local population in Guizhou Province. The minimum-spanning network showing the genetic relationships among all the 100 MLGs generated from 102 samples is presented in Figure 3A.

While some MLGs from different local populations were closely clustered together, the MSN tree showed several genotype clusters containing strains predominantly from one or select few areas. This is especially true for the BS, DZ, and PT samples (Figure 3A). For the remaining five local populations, their strains showed intermixed genotypic relationships. As expected, the geographic clustering pattern was more pronounced at the regional (in this case, provincial) level than at local population level (Figure 3B). In this analysis, the DZ population from Sichuan Province was overall well-separated from those in the other two provinces. In addition, even though there were some mixtures between the MLGs from Guizhou and Yunnan Provinces, many genotypes showed regional clustering. Together, the results are consistent with certain degree of geographic structuring in *L. hatsudake* populations in southwest China.

The MSN result indicated some host tree species-based MLG clusters. For example, four of the six strains in *Pinus kevisa* forests formed a tight cluster, and the strains in *Pinus massoniana* forests from Dazhou in Sichuan province formed two closely related clusters. However, due to the confounding factors between geography and host tree species distribution, it is impossible to distinguish whether some of the clusters were due to geographic and/or host tree species factors.

### 3.4. Genetic Differentiation Among Geographic Populations

AMOVA results indicated that the majority (60%) of the overall observed genetic variations were found among the local populations, while the remaining 40% were found within local populations (Table 5). The results are consistent with significant genetic differentiations among local populations of this species in nature. Interestingly, there was limited evidence of geographic contribution to the overall genetic variation among the three provinces beyond the local population level.

We further investigated the relationships between pairs of local populations to identify which specific local populations were genetically differentiated and which ones were similar to each other. The pairwise *Rst* values among the eight local populations are shown in Table 6. Among the eight local populations, three (i.e., the BS, DZ, and PT) were significantly differentiated from each other and from all other local populations. The largest *Rst* value was found between the local populations BS and PE (0.698), both from Yunnan province. On the other hand, several pairs of local populations, such as NJ vs. LJ, PE vs. LJ, and QN vs. LJ showed no differentiation, with an Rst value of 0.

### 3.5. Genetic Clusters and Their Geographic Distributions

**Figure 4:**
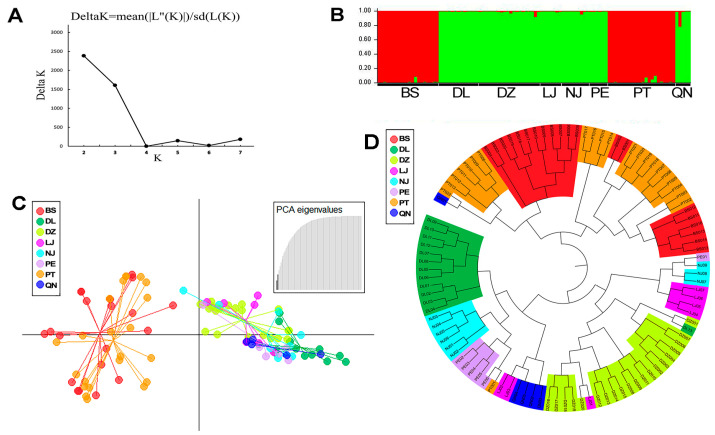
Genetic clusters of *Lactarius hatsudake* samples and their geographic distributions. (**A**) Delta K was calculated to estimate the optimal genetic population number K. (**B**) STRUCTURE ver.2.3.4 was used to assign each of the 102 samples to a genetic subpopulation and visualize the probability of belonging to each subpopulation (K = 2). Samples are arranged in a row and grouped by geographic subpopulation within the whole sample. Each color represents the isolate belonging to a different genetic subpopulation. (**C**) Genetic clustering using discriminant analysis of principal components (DAPC) of 102 samples from eight *L. hatsudake* geographic populations. (**D**) UPGMA dendrogram of eight geographic populations of *L. hatsudake* based on Nei’s genetic distance at five SSR loci.

The number of potential genetic clusters within the 102 specimens was inferred using STRUCTURE v2.3.4 based on the alleles and genotypes detected at the five marker loci. Admixture model-based simulations indicated that the most suitable K was three, with K = 2 following close behind (Figure 4A). However, both the DAPC and UPGMA clusters analyses of the 102 samples supported two clear genetic clusters, one including isolates from two local populations (BS and PT), and the other including isolates from the remaining six populations (Figure 4C,D). Interestingly, a few strains in several local populations showed some evidence for mixed ancestry.

## 4. Discussion

*Lactarius hatsudake* is an ectomycorrhizal fungus in Asia [1]. It forms symbiotic relationships with diverse pine trees and plays important roles in those ecosystems. It is also a wild edible mushroom that is widely picked and consumed in Asia, especially in southern China. Understanding its genetic diversity is fundamental to resource conservation, ecological balance, and sustainable utilization. In this study, we developed five highly polymorphic genetic markers and used them to analyze a relatively large number of fruiting bodies from diverse locations in southwest China. Our analyses revealed significant genetic differentiations among many local populations and identified evidence for heterozygous deficiency at most loci in most local populations.

The abundant allelic and genotypic diversities found in this study contrasts that reported in an earlier study based on ITS sequence. In that study (and the only study we are aware of on population genetic variations within *L. hatsudake*), limited genotypic diversity and limited evidence for genetic differentiation were found among four local populations from southern China (*Fst* = 0.0092–0.093) [17]. In addition, the potential mode of reproduction could not be inferred. The differences between our study and the previous study were likely due to several factors, including the geographic samples that were collected and the type of genetic markers that were used for analyzing them. First, only one marker locus was analyzed; thus, the discriminating power of the genotyping system was limited in the previous study. Second, although the ITS sequence has been widely used for barcoding fungal species, its variations among samples within the same species is often limited, due at least partly to its multi-copy nature in each genome and the underlying concerted evolution that can homogenize the copies, reducing its variation in the population [29]. Using the five highly polymorphic SSR markers developed here, our study identified that almost all fruiting bodies had a different MLG. Interestingly, two MLGs were shared by two fruiting bodies each, with each pair from the same local population. This result is understandable because individual genotypes of basidiomycete mushrooms can form extensive underground hyphal network, and each can produce multiple fruiting bodies aboveground, with all fruiting bodies having the same MLG. However, it is also possible that the two pairs of mushrooms had different genomes, but due to the lack of discriminating power of the five markers, our genotyping system failed to distinguish them. Additional markers including whole-genome sequencing may help distinguish these two possibilities.

Using the five highly polymorphic SSR markers, we identified that several local populations of *L. hatsudake* in southwest China showed very distinct alleles and allele frequencies. Specifically, three geographically distant local populations (Baoshan in Yunnan Province, Dazhou in Sichuan Province, and Pingtang in Guizhou Province) showed significant genetic differentiations from each other and from all the five remaining local populations. Among the remaining five local populations, variable pairs also showed significant genetic differentiations. Together, these results are consistent with geographic distance and landscape features playing a significant role in population structure of this species in southwest China. However, several geographically distant populations, including LJ (Lijiang in Yunnan Province) and QN (Qiandongnan in Guizhou Province), showed highly similar alleles and allele frequencies. At present, the reasons for such close genetic relationships between geographically distant populations are not known. Because these two sites (i.e., LJ and QN) also had different host tree species (*Pinus yunnanensis* and *Pinus massoniana*, respectively, for LJ and QN), their high genetic similarity suggested that host tree species was not a significant factor in structuring the population genetic pattern within *L. hatsudake*. This observed pattern in *L. hatsudake* (i.e., geographically distant populations being genetically highly similar) has been reported for other ectomycorrhizal mushrooms, such as *Tricholoma matsutake* and *Russula virescens* in southwest China, *Cantharellus enelensis* in eastern North America, and *Suillus luteus* in eastern South America [20,47,48,49,50,51]. However, geographical distances and barriers have significantly shaped the genetic structure of several other mushroom species. For example, wild populations of *Lentinula edodes* in China are genetically subdivided into three distinct geographical subgroups based on their geographic origins: northeast, southwest, and central China [52]. Similarly, genetic studies of *Suillus brevipes* populations across North America revealed significant genetic differentiations [53,54].

In the genetic clustering analyses based on STRUCTURE, DAPC, and UPGMA, the 102 specimens were clustered into two genetic populations (K = 2). One genetic population consisted of strains almost exclusively from two local populations, while the other cluster contained isolates from the remaining six local populations. Interestingly, both genetic clusters contained significant genetic diversity, and there was a limited relationship between geographical distance and genetic population assignments. Together, these patterns suggested that these two genetic clusters have likely diverged from each other for a long time but may have experienced recent gene flow in southwest China. The distributions of these two genetic clusters suggested both clusters have adapted to broad geographic regions and host tree species. Indeed, the three pine tree species are geographically broadly distributed, and pine trees are known to host a diversity of ectomycorrhizal fungi, such as *Tricholoma matsutake*, *Russula* spp., and *Thelephora ganbajun* [55,56,57].

In this study, the null hypothesis of HWE was rejected at all the five loci in four populations where sample sizes exceeded 10. In all cases, the observed heterozygotes were all lower than expected as generated under the hypothesis of random mating. At the same time, the genotypes at different loci were randomly associated with each other, consistent with recombination. Together, the observed heterozygous deficiency, coupled with evidence for recombination between loci, suggests that inbreeding is likely common for this species in nature. Inbreeding could be accomplished through either selfing or mating with genetically related individuals. In a tetrapolar heterothallic mushroom species, mating between germinated spores from the same fruiting body can be successful 25% of the time, while in a bipolar heterothallic species, the success rate is about 50%. Because most basidiospores from mature fruiting bodies land very close to where the fruiting body is, the chances of encountering germinated spores from the same fruiting is, thus, very high. In these matings, after one generation, the progeny of fruiting bodies would have genome-wide reduction in heterozygosity by 50% over the previous generation. At the population level, there would be a reduction in heterozygous individuals at each locus by 50%. For example, let us start with an initial population with 200 individuals. At locus A, there are two alleles A1 and A2 in equal frequency (i.e., 50% each) and the population was in HWE. Thus, the population would contain 50 A1A1, 100 A1A2, and 50 A2A2, with the heterozygous individuals A1A2 being at 50% (100/200). After one round of selfing and assuming, each individual fruiting body produced one progeny fruiting body, the progeny generation would have 75 A1A1, 50 A1A2, and 75 A2A2, a reduction in A1A2 individuals from 100 to 50 (i.e., by 50%), creating heterozygous deficiency. While the above idealized scenario of complete selfing for all individuals in a population of heterothallic mushroom, with limited outcrossing or selection, heterozygous individuals could soon become so rare that it would be difficult to detect in the population after a few generations. The presence of heterozygous individuals at all loci in all eight local populations suggested that long-distance spore dispersal and outcrossing have contributed to the maintenance of genetic diversity and potential adaptation in *L. hatsudake* populations in southwest China [14,58].

In species capable of outcrossing, inbreeding is often linked to lower fitness among progeny than those from outcrossing [59]. The signature of inbreeding in a population is a lower observed heterozygosity than expected under random mating. The phenomenon of lower than expected heterozygosity has also been found in other edible mushrooms, such as *Boletus edulis* and *Lentinula edodes*, and a deadly poisonous mushroom, *Trogia venenata* [60,61,62]. In contrast, an observed higher than expected heterozygosity has been reported in both edible and poisonous mushrooms, like *Agaricus bisporus, Amanita exitialis*, and *Flammulina filiformis* [59,63,64,65]. Based on the classical heterosis theory, higher observed heterozygosity is often associated with enhanced fitness, and such a principle has been used for breeding hybrid cultivars of many crops, including the commercial button mushroom *A. bisporus* [66,67]. However, by exposing deleterious alleles in homozygous states frequently, inbreeding and selection could also facilitate the elimination of deleterious alleles from the population. Long-term fine-scale monitoring is required to understand the relative importance of inbreeding and outcrossing for population genetic structure and the adaptation of this species in nature.

## 5. Conclusions

This study represents the first comprehensive investigation of the genetic diversity and population structure of the wild edible ectomycorrhizal mushroom *L. hatsudake*. By developing five high-resolution SSR markers and analyzing eight natural populations, we uncovered substantial genetic diversity and differentiation in southwest China. A total of 100 unique MLGs were identified, with most of the observed genetic variation contributed by between-populations factors rather than within individual local populations. This pattern highlights the importance of geographical isolation on the distributions of alleles and genotypes. Notably, two distinct genetic clusters were detected, suggesting historical divergence within this species. Interestingly, the distributions of the two genetic subpopulations showed no clear geographical, altitudinal, and host plant associations, which might reflect the species’ strong dispersal and adaptive capacities, counterbalance the physical barriers in this fragmented mountainous landscape in southwest China. However, anthropogenic influences, such as habitat modification or unintentional spore transport by mushroom collectors and consumers, and limitations inherent to certain molecular markers may also contribute to the observed pattern of genetic variations. We propose that the observed heterozygote deficiency was likely due to prevalent inbreeding for *L. hatsudake* in nature. These findings advance our understanding of *L. hatsudake*’s evolutionary history and ecological adaptation in southwest China’s variable geography and dynamic environment. Future studies should prioritize whole-genome sequencing approaches to understand the genetic bases of fungi–host plant interactions, local adaptation, and reproductive barriers.

## Figures and Tables

**Table 1:** Information of *Lactarius hatsudake* sample collection sites in southwest China.

Code	City	Province	Sample Size	Altitude (Meters Above Sea Level)	Main Host Plant
BS	Baoshan	Yunnan	20	1200–1600 m	*Pinus massoniana*
DL	Dali	Yunnan	13	2100–2600 m	*Pinus yunnanensis*
DZ	Dazhou	Sichuan	20	800–1200 m	*Pinus massoniana*
LJ	Lijiang	Yunnan	7	2600–3200 m	*Pinus yunnanensis*
NJ	Nujiang	Yunnan	9	1500–2700 m	*Pinus yunnanensis*
PE	Pu’er	Yunnan	6	1200–1500 m	*Pinus kesiya*
PT	Pingtang	Guizhou	22	600–1500 m	*Pinus massoniana*
QN	Qiandongnan	Guizhou	5	1500–1600 m	*Pinus massoniana*
Total			102		

**Table 2:** Information about the five simple sequence repeat markers developed and used for genotyping fruiting bodies of *Lactarius hatsudake* from southwest China.

ID	Repeat Motif	Forward Primer (5′-3′)	Tm	Reverse Primer (5′-3′)	Tm	Contig	Nucleotide Span	Dye Label
*Lh1*	CA	GCCAAATTTGACATCGCACG	59.02	ACGGAGATACAACGAGGCAA	59.11	054	321,916–322,114	6-FAM
*Lh4*	GT	GGCAGTGGTCCATTTGTGAG	59.12	TTGACTTCCCACAG-CTTTGC	58.97	359	34,257–34,451	6-FAM
*Lh5*	TTA	CCGTGCCGTTCTTCC-AATAG	58.99	CCAAATCACATTAGGCCACCA	58.54	034	195,359–195,544	6-FAM
*Lh8*	GGT	ATGTCCCAAACCCA-ACAAGC	58.74	TCCTAGTCGTCCATTTCCCC	58.97	159	38,015–38,224	6-FAM
*Lh14*	AG	TGGAAAGTTGGTCGACGGTA	58.96	AGCACCAAATCCGCCTAGT	59.01	062	52,079–52,321	6-FAM

**Table 3:** Mean number of alleles and allelic diversity (±standard error) at the five SSR loci within eight local populations of *Lactarius hatsudake* in southwest China.

Population	*Na*	*Ne*	*uH*
BS	5.600 ± 1.208	3.497 ± 0.889	0.674 ± 0.062
DL	7.200 ± 1.200	3.990 ±0.800	0.729 ± 0.067
DZ	7.400 ± 1.288	4.669 ± 0.721	0.781 ± 0.041
LJ	5.800 ± 0.663	4.132 ± 0.776	0.782 ± 0.048
NJ	6.000 ± 0.548	4.236 ± 0.316	0.803 ± 0.021
PE	6.000 ± 1.049	4.145 ± 1.002	0.748 ± 0.088
PT	6.400 ± 1.122	4.108 ± 0.936	0.720 ± 0.061
QN	5.000 ± 0.447	3.738 ± 0.228	0.809 ± 0.021

*Na* = number of unique alleles; *Ne* = effective number of alleles; *uH =* unbiased allelic diversity.

**Table 4:** Chi-square tests for Hardy–Weinberg equilibrium within eight local populations of *Lactarius hatsudake* in southwest China.

Population	Locus	Observed Heterozygotes	Expected Heterozygotes	HWE Chi-Square Value
BS	Lh1	0	13.700	100.000 ***
	Lh4	12	17.125	102.117 ***
	Lh5	4	13.688	60.408 ***
	Lh8	0	9.700	40.000 ***
	Lh14	1	12.075	40.408 ***
DL	Lh1	0	10.000	65.000 ***
	Lh4	2	11.000	91.520 ***
	Lh5	8	10.154	81.725 *
	Lh8	4	6.462	26.642 **
	Lh14	4	7.923	21.031 *
DZ	Lh1	1	16.125	120.118 ***
	Lh4	0	13.100	60.000 ***
	Lh5	20	17.988	111.190 **
	Lh8	1	13.475	60.089 ***
	Lh14	20	16.500	122.061 ***
LJ	Lh1	2	6.000	42.778 *
	Lh4	1	5.214	21.280 *
	Lh5	6	6.607	22.286
	Lh8	4	5.286	23.030
	Lh14	1	4.071	21.000 **
NJ	Lh1	2	6.778	38.469 ***
	Lh4	2	7.222	45.360 **
	Lh5	5	6.889	22.360
	Lh8	5	7.056	16.650
	Lh14	1	6.617	18.184 **
PE	Lh1	4	5.167	42.667
	Lh4	5	4.917	30.000
	Lh5	3	4.417	12.000
	Lh8	2	2.500	12.074
	Lh14	1	3.583	12.122
PT	Lh1	0	18.182	154.000 ***
	Lh4	0	18.818	198.000 ***
	Lh5	3	11.932	46.444 ***
	Lh8	2	14.091	44.220 ***
	Lh14	2	14.409	44.321 ***
QN	Lh1	1	3.700	20.000*
	Lh4	1	3.300	10.200
	Lh5	3	3.800	16.250
	Lh8	2	3.800	25.000 *
	Lh14	0	3.600	15.000 *

Bold chi-square values indicate that the hypothesis of random mating was rejected, *p* < 0.05 (*), <0.01 (**) and <0.001(***).

**Table 5:** Two-level hierarchical AMOVA of *Lactarius hatsudake* in southwest China.

Source	df	SS	MS	Est. Var.	% Contribution
Among Regions	2	7954.176	3977.088	0.000	0
Among Local Pops	5	18,778.340	3755.668	353.815	60.32 ***
Within Local Pops	94	21,879.955	232.765	232.765	39.68 ***
Total	101	48,612.471		586.581	100

SS: sum of squared values; MS: mean squared values; ***, *p* < 0.001.

**Table 6:** Pairwise comparisons of genetic differentiation among the eight *Lactarius hatsudake* local populations in southwestern China.

	BS	DL	DZ	LJ	NJ	PE	PT	QN
BS	0.000							
DL	0.599 ***	0.000						
DZ	0.465 ***	0.408 ***	0.000					
LJ	0.621 ***	0.017	0.394 ***	0.000				
NJ	0.604 ***	0.033	0.336 ***	0.000	0.000			
PE	0.698 ***	0.175 ***	0.504 ***	0.000	0.026	0.000		
PT	0.168 ***	0.380 ***	0.418 ***	0.413 ***	0.385 ***	0.505 ***	0.000	
QN	0.560 ***	0.102 *	0.314 ***	0.000	0.000	0.000	0.333 ***	0.000

Full names of local population abbreviations are shown in Table 1. *, *p* < 0.05; ***; *p* < 0.001.

## Data Availability

The original contributions presented in this study are included in the article/Supplementary Materials. Further inquiries can be directed to the corresponding authors.

## Supplementary Materials

The following supporting information can be downloaded at: https://www.mdpi.com/article/10.3390/jof11060438/s1, Table S1: Multilocus genotypes of Lactarius hatsudake from southwest China.
